# Histopathological aspects of the relationship *Saimiri sciureus* × *Prosthenorchis elegans* (Acanthocephala) in a preserved environment of an urban rainforest fragment

**DOI:** 10.1590/S1984-29612025013

**Published:** 2025-03-31

**Authors:** Rogério Antonio Ribeiro Rodrigues, Washington Luiz Assunção Pereira, Raul Henrique da Silva Pinheiro, Elane Guerreiro Giese

**Affiliations:** 1 Programa de Pós-graduação em Saúde e Produção Animal na Amazônia, Instituto da Saúde e Produção Animal, Universidade Federal Rural da Amazônia – UFRA, Belém, PA, Brasil; 2 Laboratório de Patologia Animal, Instituto da Saúde e Produção Animal, Universidade Federal Rural da Amazônia – UFRA, Belém, PA, Brasil; 3 Laboratório de Histologia e Embriologia Animal, Instituto da Saúde e Produção Animal, Universidade Federal Rural da Amazônia – UFRA, Belém, PA, Brasil

**Keywords:** Helminths, intestinal parasite, Squirrel monkeys, neotropical primates, Amazon, Helmintos, parasitos intestinais, macaco de cheiro, Primatas neotropicais, Amazônia

## Abstract

Non-human primates are potential hosts for helminths of various phyla, and the presence of these parasites can cause changes in organ morphology and functionality. In this study, we present the alterations induced by the presence of acanthocephalan parasites in the small intestine of a snub-nosed monkey that died in the Bosque Rodrigues Alves, municipality of Belém, Pará state, Brazil. Ten specimens of *Saimiri sciureus* had their intestines analyzed and the parasites recovered were cleaned, quantified, fixed and observed using light microscopy and scanning electron microscopy, and fragments of the intestine were separated for histological analysis to identify the alterations. All the animals were parasitized, and a total of 50 specimens representing the Acanthocephala Phylum were recovered, which morphologically showed characteristics compatible with *Prosthenorchis elegans* (Diesing, 1851). Light microscopy revealed the presence of parasites attached to the mucosal layer, passing through the underlying layers until they reached the muscular layer. Scanning electron microscopy showed the tissue lesions caused by the proboscis hooks and the flattening of the intestinal villi in the presence of the parasite. The presence of the parasites in the intestine changed the morphology of the organ, possibly causing loss of functionality at the site of attachment and adjacent tissue.

## Resumo

Os primatas não humanos são potenciais hospedeiros de helmintos de diversos filos. A presença desses parasitos pode ocasionar alterações na morfologia e funcionalidade dos órgãos. Neste estudo, foram apresentadas as alterações induzidas pela presença de acantocéfalos parasitos do intestino delgado de macaco de cheiro que vieram a óbito no Bosque Rodrigues Alves, município de Belém, Pará. Dez exemplares de *Saimiri sciureus* tiveram o intestino analisado, e os parasitos recuperados foram limpos, quantificados, fixados e observados por microscopia de luz e microscopia eletrônica de varredura, bem como os fragmentos do intestino foram separados para análise histológica. Todos os animais estavam parasitados, sendo recuperado um total de 50 exemplares representantes do Filo Acanthocephala, que morfologicamente apresentavam características compatíveis a *Prosthenorchis elegans* (Diesing, 1851). Em microscopia de luz, foi possível observar a presença dos parasitos fixados na camada mucosa transpassando as camadas subjacentes até alcançar a camada muscular. Em microscopia eletrônica de varredura foi possível observar as lesões teciduais, causadas pelos ganchos da probóscide e o achamento das vilosidades intestinais na presença do parasito. A presença dos parasitos no intestino modificou a morfologia do órgão, possivelmente ocasionando perda da funcionalidade no local de fixação e tecido adjacente.

## Introduction

Primates are considered important reservoirs of infectious and parasitic diseases (Sobreira et al., 2020). The diversity of parasitic agents in primates includes acanthocephalans from the genus *Prosthenorchis* (Rudolphi, 1819), made up of 20 species, all of which are parasites of mammals (Machado, 1950), with *Prosthenorchis spirula* (Olfers, 1819) and *Prosthenorchis elegans* (Diesing, 1851) being the most commonly recorded species (Machado, 1950; Pissinatti et al., 2007; Catenacci et al., 2016; Oliveira et al., 2017; Zárate-Ramos et al., 2018; Rojas-Sánchez et al., 2022, 2023).

Parasite infections can be associated with different predisposing factors such as stress, immunosuppression or even a previously contaminated environment (Pereira et al., 2020). The adult helminths of *Prosthenorchis* colonize the host's intestine, adhering firmly to the tissue and cause lesions in the epithelium and in the underlying layers up to the intestinal musculature, which impair the action of that organ and can cause the death of the hosts (Dunn, 1963; Pissinatti et al., 2007; Oliveira et al., 2017; Rojas-Sánchez et al., 2023).

In view of the above, the importance of making an inventory of the helminth fauna of non-human primates in Brazil is clear. Therefore, this study aims to describe the main aspects of the parasite-host relationship caused by the presence of *Prosthenorchis elegans* in the intestine of *Saimiri sciureus* and the histological changes induced by the presence of this parasite.

## Materials and Methods

### Origin of the samples

The samples used were donated by Bosque Rodrigues Alves Jardim Botânico da Amazônia, Belém-PA (1°25'44“S, 48°27'40”W) to the Animal Pathology Laboratory of the Federal Rural University of Amazonia (LABOPAT/UFRA), which received 14 specimens of *Saimiri sciureus* that had died suddenly in the environment where they lived.

Of this total, four specimens were at an advanced state of decomposition and there were cadaveric phenomena that made it impossible to carry out anatomopathological examinations and obtain samples for complementary tests. As a result, ten specimens of *S. sciureus* were submitted for necroscopic examination to determine the cause of death. Specimens of Acanthocephala were recovered from the small intestine, fixed, quantified and identified. The taxonomic classification of Acanthocephala was in accordance with Machado (1950).

### Sample preparation and processing

Fragments of the intestine were cleaved and fixed in 10% formaldehyde and then subjected to routine histological techniques. The acanthocephalans were separated into Petri dishes and analyzed under light microscopy.

For scanning electron microscopy, four specimens were washed in distilled water, postfixed in 1% osmium tetroxide, dehydrated to the critical point of CO2, metallized with gold + palladium, and analyzed using a TESCAN scanning electron microscope (VEGA 3) in the Laboratório de Microscopia Eletrônica de Varredura, Universidade Federal Rural da Amazônia.

## Results

**Figure 1:**
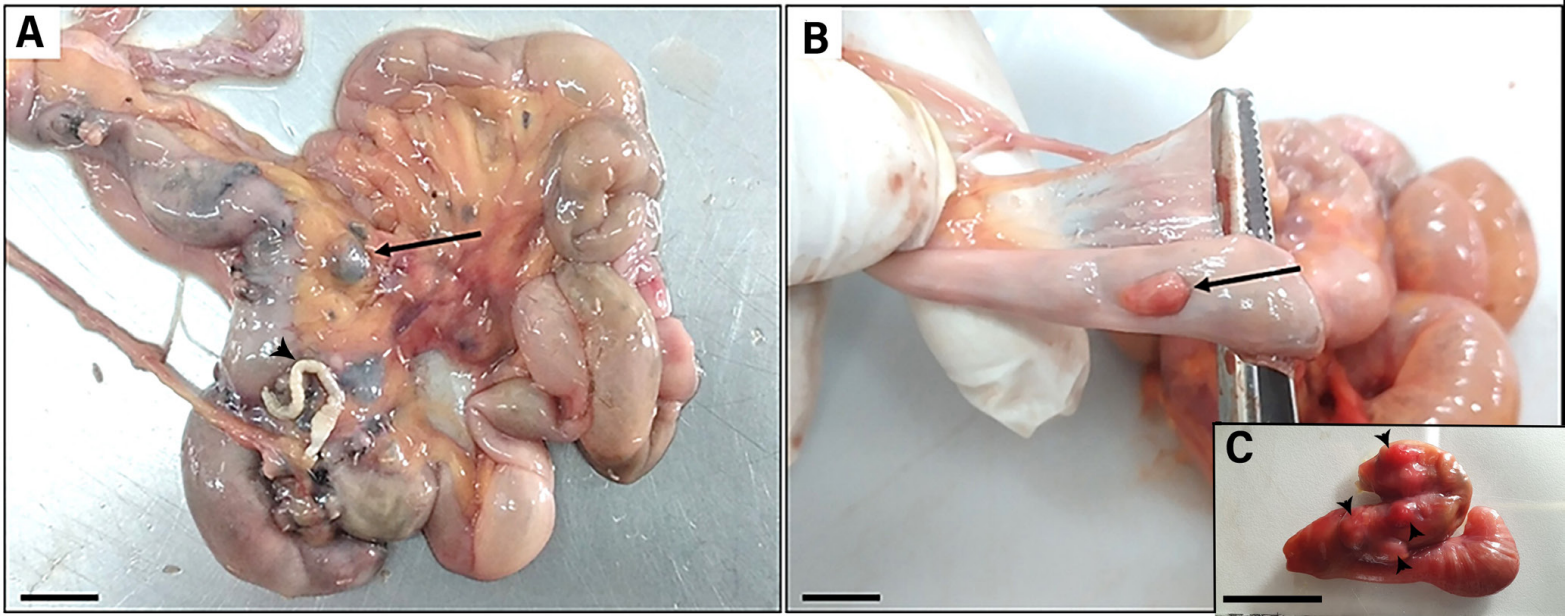
Photomacrograph of the intestine of *Saimiri sciureus*. (**A**) shows reactive lymph nodes in the mesentery of the host (arrows) and acanthocephalans (arrowheads); (**B, C**) detail the presence of nodules of firm consistency and reddish coloration in the serous layers of the intestine parasitized by acanthocephalans (arrow and arrowheads). Scale bars: 1 cm, 1cm, 2 cm, respectively.

All the animals observed in this study were parasitized by robust acanthocephalans that were anchored in the mucous membranes of the ileum and caecum and sometimes partially obstructed their lumen. Externally, it was possible to observe reactive lymph nodes in the mesentery of some hosts (Figure 1A). It was also possible to confirm the presence of nodules with a firm consistency and a reddish color in the serous layers of the intestines parasitized (Figures 1B, C).

**Figure 2:**
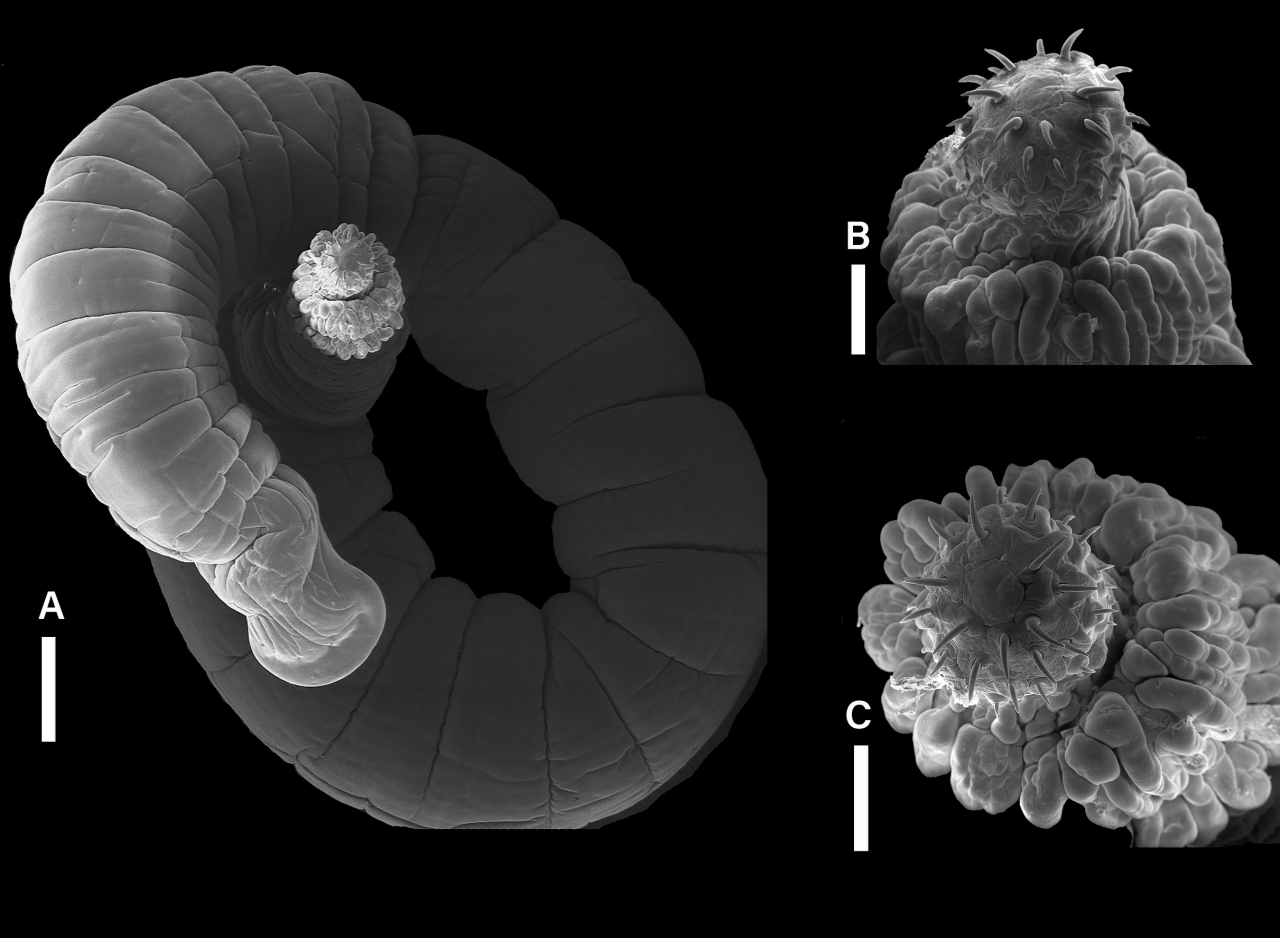
Scanning electron microscopy of *Prosthenorchis elegans*. (**A**) shows the general view of the helminth with a horseshoe-shaped posterior end. (**B**) and (**C**) it is possible to see the details of the anterior end of the helminth, in which the proboscis of the helminth can be seen 4-5 rows of 6 hooks and the cephalic collar formed by the folds of the integument immediately below the proboscis. Scale bar: 1 mm, 200 µm and 200 µm, respectively.

A total of 50 helminths (26 males and 34 females) were recovered from the small intestines of ten specimens of *Saimiri sciureus*, showing a prevalence of 100% (10 infected, 10 examined), mean intensity: 5, mean abundance: 5 and amplitude: 3 to 20 parasites per animal. All the specimens collected showed characteristics compatible with representatives of the Phylum Acanthocephala, genus *Prosthenorchis*, and morphology compatible with *Prosthenorchis elegans* (Figure 2A).

Morphologically, the specimens of *Prosthenorchis elegans* showed proboscises with 6 rows of 4-5 hooks, each arranged obliquely, folds in the integument forming a collar-like structure (Figures 2B, C). In addition, the posterior end of the females of this species has a shallow depression, forming a lateral stranglehold on the tail, which takes on the shape of horseshoe-shaped (Figure 2A).

**Figure 3:**
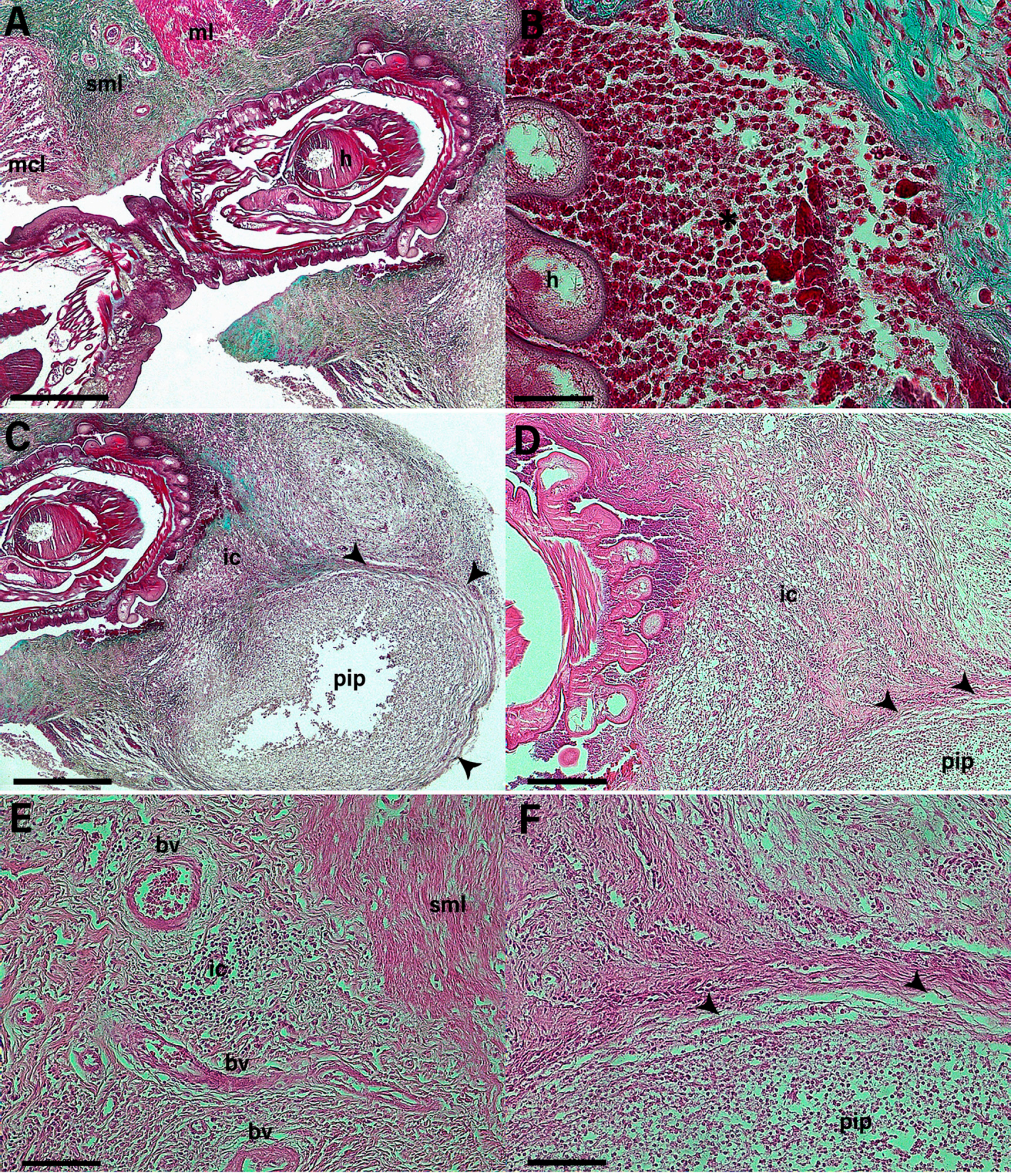
Photomicrograph of the intestinal segment of *Saimiri sciureus* parasitized by *Prosthenorchis elegans*. (**A**) we can see the helminth (**h**) anchored to the intestinal segment, passing through the mucosal (**mcl**) and submucosal (**sml**) layers and reaching the muscular layer (**ml**). (**B**), we can see the eosinophilic inflammatory process (*) near the helminth (**h**) anchorage. (**C**) and (**D**) show the pyogenic inflammatory process (**pip**), with connective tissue (arrowhead) surrounding the inflammatory process, and massive inflammatory cells (**ic**) can be seen towards the anchoring site. (**E**) and (**F**), the presence of inflammatory cells (**ic**) can be seen in the submucosal layer, where dilated blood vessels (**bv**) and the proliferation of connective tissue (arrowhead) surrounding the pyogenic inflammatory process (**pip**) can be seen. (A), (B) and (C): Gomori trichrome stain (Light Green). (D), (E) and (F): Hematoxylin and Eosin (HE) staining. Scale bar: 500 µm, 50 µm, 500 µm, 200 µm, 100 µm and 100 µm respectively.

Light microscopy analysis showed that the acanthocephalans were anchored to the mucosal layer of the parasitized organ. In some cases, the helminths penetrated the other layers of the intestines until they reached the muscle layer (Figure 3A). At the site of the helminth's anchorage, there was intense migration of inflammatory cells, made up mainly of eosinophils, concentrated around the proboscis attached to the tissue (Figure 3B).

The presence of the helminths led to the beginning of a pyogenic inflammatory process, showing proliferation of connective tissue, involving and isolating a collection of integrated and degenerated inflammatory cells, with the presence of amorphous content in the center of the lesion (Figures 3C, D). There was also a change in the vascularization adjacent to the inflammatory process, especially in the submucosal layer of the parasitized intestine, where granulation tissue and dilated blood vessels containing red blood cells were observed (Figures 3E, F).

**Figure 4:**
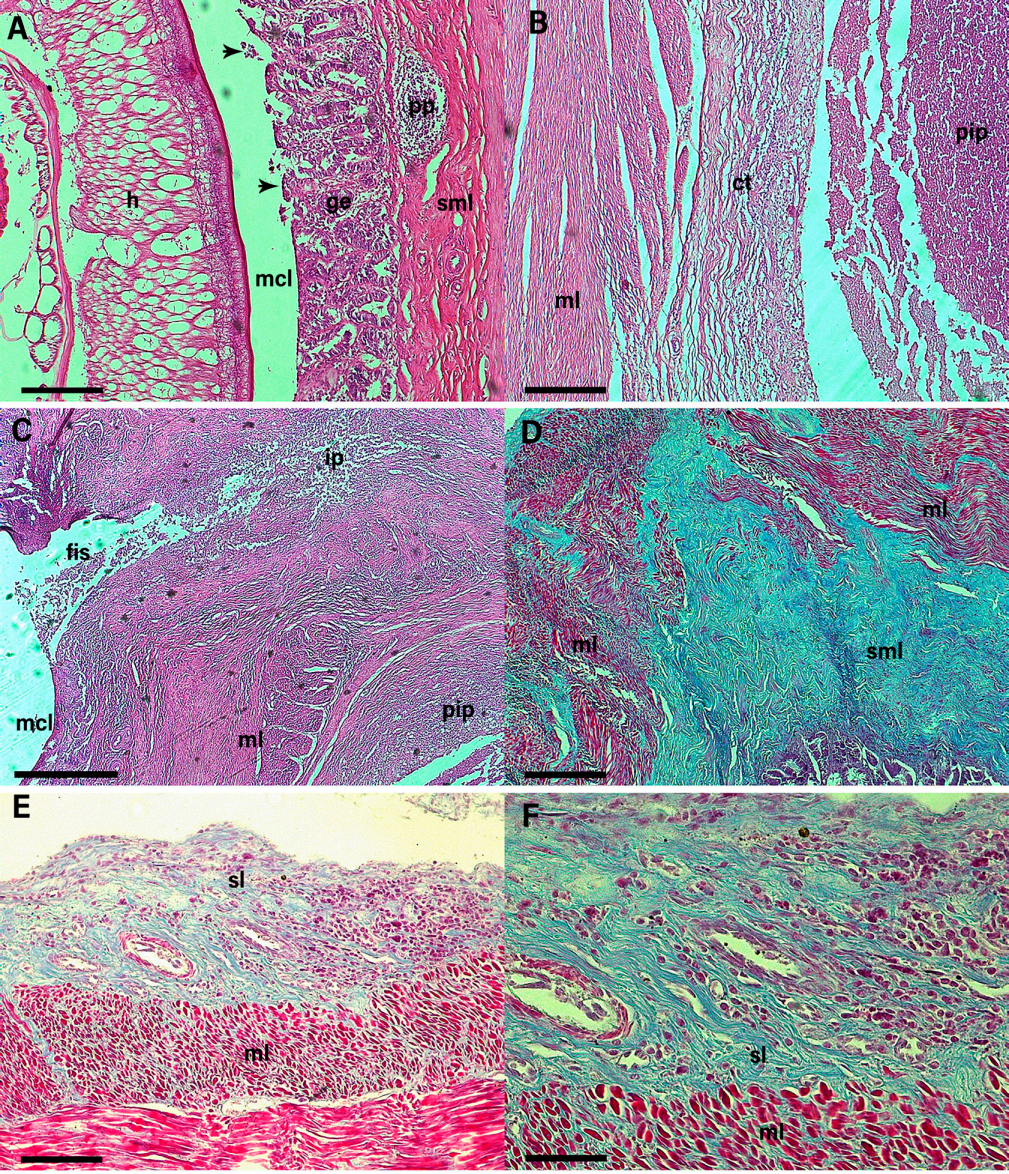
Photomicrograph of the intestinal segment of *Saimiri sciureus* parasitized by *Prosthenorchis elegans*. (**A**) we can see the body of the helminth (**h**) present in the mucosal layer (**mcl**), crushing and destroying the intestinal villi, damaging the absorptive epithelium (arrowhead) and glandular epithelium (**ge**), inducing the manifestation of Peyer's plaques (**pp**) in the submucosal layer (**sml**). (**B**) the formation of the pyogenic inflammatory process (**pip**) stands out, surrounded by connective tissue (**ct**), underlying the muscular layer (**ml**) of the intestine. (**C**) shows the presence of a fistula (**fis**) connecting the mucous layer (**mcl**) to the pyogenic inflammatory process (**pip**) present in the inner layers of the intestine. (**D**) we can see the connective tissue present in the submucosal layer (**sml**) infiltrating between the muscle fibers of the muscular layer (**ml**). (**E**) and (**F**) show the presence of an eosinophilic inflammatory infiltrate in the serous layer (**sl**) near the muscular layer (**ml**) of the parasitized intestine. (A), (B) and (C): Hematoxylin and Eosin (HE) staining. (D), (E) and (F): Gomori Trichrome (Light Green). Scale bar: 200 µm, 200 µm, 500 µm, 200 µm, 100 and 50 µm respectively.

Atrophy and reduction of the intestinal villi was observed due to the presence and compression exerted by the helminth on the intestinal mucosa and, consequently, a reduction in the lining epithelium as well as the glandular epithelium of the organ (Figure 4A). A fistula was identified next to the pyogenic inflammatory process, which connected the lesions of the underlying layers to the mucous layer and acted as a gateway for continuous bacterial contamination and maintenance of the chronic-active inflammatory processes (Figures 4B, C).

The presence of dense connective tissue infiltrating the injured muscle fibers was observed due to the continuous lesions in the muscle layer of the parasitized organ, which suggests the occurrence of a healing process in this layer. It was possible to see the presence of an inflammatory infiltrate in the submucosal and serosal layers (Figures 4D, E, F).

**Figure 5:**
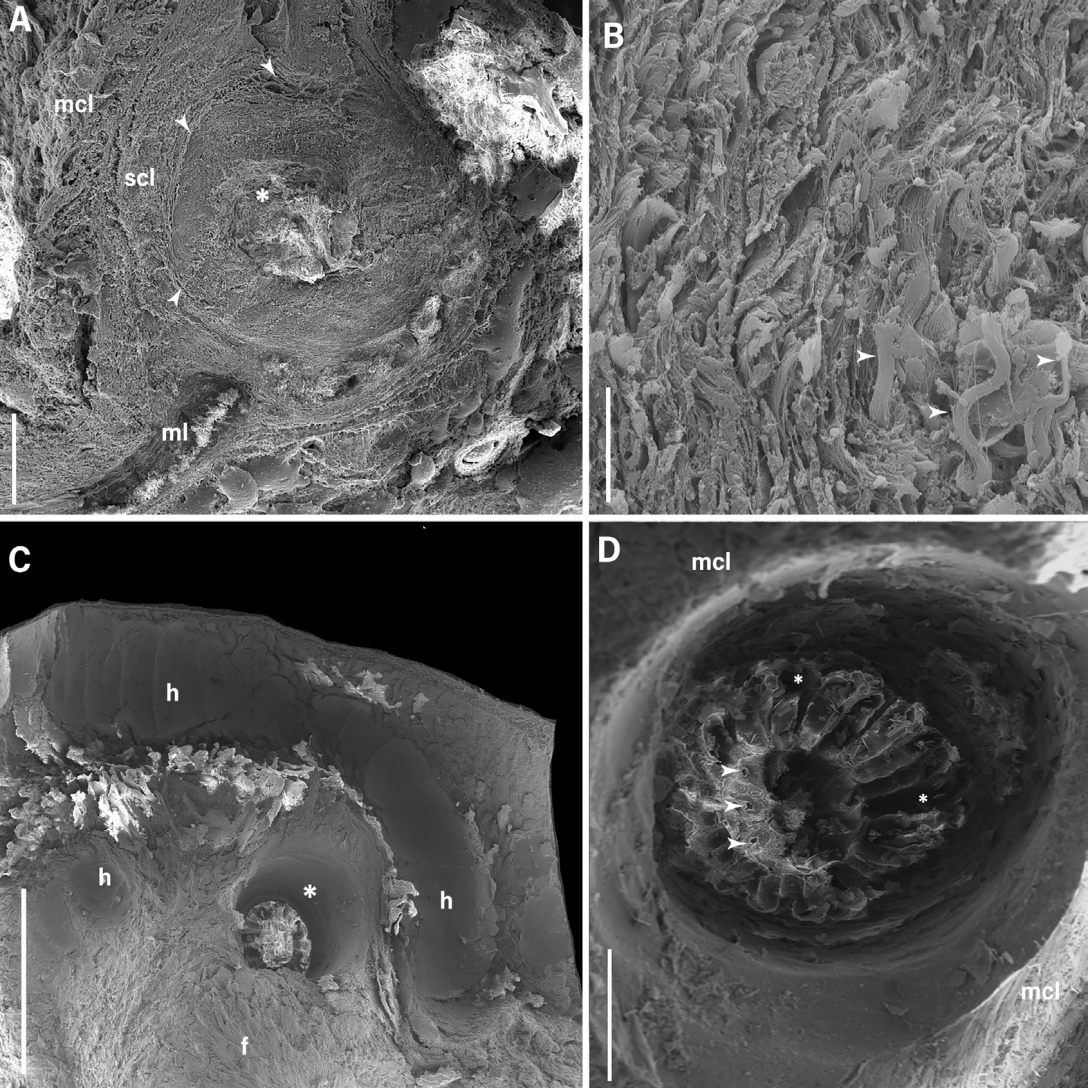
Scanning electron microscopy of the details of the lesions induced by *Prosthenorchis elegans* in the intestine of *Saimiri sciureus*. (**A**) details the formation of the pyogenic inflammatory process, which was formed below the mucosal layer (**mcl**), lying predominantly between the submucosal (**scl**) and muscular layers (ml), with connective tissue (arrowhead) surrounding the amorphous content inside (*). (**B**) highlights the proliferation of dense connective tissue (arrowhead) in response to the ongoing inflammatory process. (**C**) highlights the flattening (**f**) of the intestinal villi due to compression by the helminth's body (**h**) and the ulcerative lesions caused at the anchoring site (*). (**D**) highlights the details of the lesions due to the helminth's anchoring in the mucosal layer of the intestine (**mcl**), where it is possible to see the markings of the cephalic collar (*) and the holes caused by the helminth's hooks (arrowhead). Scale bar: 500 µm, 10 µm, 2 mm and 500 µm respectively.

The tissue lesions were observed in layers. It was clear that the pyogenic inflammatory process was present between the submucosal and muscular layers. It was also possible to elucidate and delimit this reaction, which showed proliferation of connective tissue distributed in a circular and peripheral fashion, surrounding and isolating the collection of whole inflammatory cells and bacteria present inside this lesion, with centralized amorphous content (Figures 5A, B).

It was possible to observe the lesions that the helminth induced when it anchored itself to the segment of intestinal mucosa. That made it possible to pinpoint the ulcerative lesions on the mucosal surface, indicating the anchoring sites and the perforations caused by the hooks of the acanthocephalans. Flattening of the intestinal villi and intense epithelial exfoliation on the mucosal layer were visualized (Figures 5C, D).

## Discussion

The occurrence of *Prosthenorchis elegans* in non-human primates has been reported in different countries on the American continent: Costa Rica (Rojas-Sánchez et al., 2022, 2023), Mexico (Zárate-Ramos et al., 2018), and also in Brazil (Pissinatti et al., 2007; Oliveira et al., 2017; Soares et al., 2024). In this study, the prevalence was 100%, demonstrating an intrinsic parasite-host relationship and maintenance of its life cycle. Primates act as definitive hosts, while invertebrates such as cockroaches (Blattodea) and beetles (Coleoptera) are intermediate hosts for *Prosthenorchis elegans* (Bowman, 2010). Although high infection rates did not occur in this study, according to Oliveira et al. (2017) the chronic evolution of parasitism can be characterized by watery diarrhea for several months, with weakness and progressive weight loss of the animals. The acute manifestation of the disease occurs when the parasite perforates the intestinal wall, leading to bacterial peritonitis and sepsis (Pissinatti et al., 2007; Müller et al., 2010).

Because they are robust and large, *P. elegans* specimens, when present in the intestines, are responsible for severe morphological changes in all layers of the host intestines. The studies by Oliveira et al. (2017), Rojas-Sánchez et al. (2022, 2023) showed that the serous layers of the intestines of the hosts they analyzed had nodulations that were perceptible to the naked eye. These changes were also observed in 100% of the specimens analyzed in this study, and possibly occurred as a result of the underlying inflammatory reactions that occurred concomitantly with the inflammatory processes underway during parasitosis.

In this study, the robust nature of the parasites found in the intestines of *S. sciureus* caused the lumen of the intestine to close, and was responsible for causing morphological changes in all layers of the intestines. Pissinatti et al. (2007), when analyzing the intestine of other non-human primates, *Leontopithecus chrysomelas,* under light microscopy, observed that the organ had multiple parasites attached to the mucosa, causing ulcerative enteritis and triggering the migration of various inflammatory cells to the area of infection.

Oliveira et al. (2017) reported the installation of a diffuse inflammatory process from the mucosal layer to the submucosal layer, and proliferation of fibroblasts and collagen and neovascularization in *Callithrix geofroyi*. Rojas-Sánchez et al. (2023) reported similar findings, adding that this type of reaction occurred between the muscular and serous layers, with the presence of macrophages, neutrophils, eosinophils, fibroblasts and lymphocytes in *Saimiri oerstedii citrinellus*.

In the present study, it was possible to identify that this productive inflammatory process occurred between the submucosal and muscular layers, revealing a proliferation of connective tissue, which surrounded and isolated the predominantly whole and degenerated pyocyst cells, as well as bacteria in an amorphous content at the center of the lesion, unlike the reports above. This pathological condition manifested itself in the vicinity of the helminth's anchoring region as a result of the constant injuries caused to the parasitized organ. Although the studies by Oliveira et al. (2017), Rojas-Sánchez et al. (2023) and the present study identified the inflammatory process in different layers of the parasitized organ, these severe inflammatory responses were caused primarily by *P. elegans* and represent a high degree of harmfulness to the host.

The presence of *P. elegans* in the intestinal mucosa caused flattening of the intestinal villi, which reduced the range of their projections and compromised the function of the parasitized organ. This finding was not reported by Pissinatti et al. (2007), Oliveira et al. (2017) and Rojas-Sánchez et al. (2023). However, this lesion tends to occur due to massive parasitism and the size of the helminths. Furthermore, in the present study, the ulcerative lesions caused by the proboscis hooks in the intestinal mucosa probably produced entry points for bacteria to invade and colonize the underlying layers of the intestine, resulting in fistulae in the intestines of *S. sciureus*.

Although not previously reported, this is a relevant clinical finding since this type of lesion perpetuates the underlying suppurative processes and worsens the clinical condition of the hosts, which may manifest as apathy, inappetence, abdominal pain, diarrhea, progressive weight loss and death, as reported by Pissinatti et al. (2007) and Rojas-Sánchez et al. (2023), in addition to indicating varying degrees of lesions.

With regard to possible treatments, there are few studies along these lines. Zárate-Ramos et al. (2018) proposed an alternative for captive animals in Mexico. These authors suggest the use of loperamide-niclosamide hydrochloride (Lomotilt Pfizer) at a dose of 0.5 mg/0.9 kg for 3 days. However, the authors report that this treatment only eliminated the young forms, and surgical procedures are necessary for removing the adult parasites.

## Conclusion

The present study showed that the presence of *P. elegans* induced significant histopathological changes in the morphology of the host intestine. These affected all layers of this organ, inducing destruction of the absorptive epithelium, reduction of intestinal villi, massive migration of inflammatory cells to the mucosal, submucosal, muscular and serous layers, affecting the morphology and altering the function of the organ, thus causing lesions in all layers and inducing the reparative proliferation of connective tissue between the muscle fibers of the muscular layer of the parasitized intestine.

## References

[B001] Bowman DD, Bowman DD (2010). Georgis parasitologia veterinária..

[B002] Catenacci LS, Colosio AC, Oliveira LC, De Vleeschouwer KM, Munhoz AD, Deem SL (2016). Occurrence of *Prosthenorchis elegans* in Free-living Primates from the Atlantic Forest of Southern Bahia, Brazil. J Wildl Dis.

[B003] Dunn FL (1963). Acanthocephalans and cestodes of South American monkeys and marmosets. J Parasitol.

[B004] Machado DA (1950). Revisão do gênero *Prosthenorchis* Travassos, 1915 (Acanthocephala). Mem Inst Oswaldo Cruz.

[B005] Müller B, Mätz-Rensing K, Pérez Yamacita JG, Heymann EW (2010). Pathological and parasitological findings in a wild red titi monkey, *Callicebus cupreus* (Pitheciidae, Platyrrhini). Eur J Wildl Res.

[B006] Oliveira AR, Hiura E, Guião-Leite FL, Flecher MC, Braga FR, Silva LP (2017). Pathological and parasitological characterization of *Prosthenorchis elegans* in a free-ranging marmoset *Callithrix geofroyi* from the Brazilian Atlantic Forest. Pesq Vet Bras.

[B007] Pereira FV, Lucena FP, Rodrigues RL, Barros LA, Pires CA, Ferreira AMR (2020). Prevalência e distribuição espacial da ocorrência de helmintos em primatas não humanos de vida livre no estado do Rio de Janeiro, Brasil. Arq Bras Med Vet Zootec.

[B008] Pissinatti L, Pissinatti A, Burity CHF, Mattos DG, Tortelly R (2007). Ocorrência de Acanthocephala em *Leontopithecus* (Lesson, 1840), cativos: aspectos clínico-patológicos. Callitrichidae-Primates. Arq Bras Med Vet Zootec.

[B009] Rojas-Sánchez E, Umaña‑Blanco F, Jiménez‑Rocha A, Vega‑Benavides K, Medaglia A, Solano‑Barquero A (2023). Cryptic diversity in a gastrointestinal acanthocephalan of New World primates from Costa Rica. Sci Rep.

[B010] Rojas-Sánchez E, Vega-Benavides K, Jiménez-Rocha AE, Rodriguez-Dorado E, Jimenez-Soto M (2022). Medical-surgical management of intestinal infection by *Prosthenorchis elegans* in nonhuman primates from Costa Rica. J Zoo Wildl Med.

[B011] Soares ADS, Silva MB, Fraga RE, Hoppe EGL, Oliveira WJ, Schiavetti A (2024). Helminths of Wied’s marmoset (*Callithrix kuhlii* (Coimbra-Filho, 1985) (Primates: Callitrichidae)) from the Atlantic Forest, Southern Bahia State, Brazil. Rev Bras Parasitol Vet.

[B012] Sobreira EA, Lage VS, Lage RS, Lage GS, Krahl G. (2020). Estudos em zootecnia e ciência animal..

[B013] Zárate-Ramos JJJ, Gómez-Garza MA, Rodríguez-Tovar LE, Hernández-Escareno J, Contreras-Lozano JA (2018). An Alternative Treatment Against Acanthocephala (*Prosthenorchis elegans*) in Captive Squirrel Monkeys (*Saimiri sciureus*) in Mexico. J Parasitol.

